# Metabolic Alterations in Sepsis

**DOI:** 10.3390/jcm10112412

**Published:** 2021-05-29

**Authors:** Weronika Wasyluk, Agnieszka Zwolak

**Affiliations:** 1 Chair of Internal Medicine and Department of Internal Medicine in Nursing, Faculty of Health Sciences, Medical University of Lublin, 20-093 Lublin, Poland; agnieszka.zwolak@umlub.pl; 2 Doctoral School, Medical University of Lublin, 20-093 Lublin, Poland

**Keywords:** sepsis, septic shock, critical illness, intensive care, metabolism, metabolic disorders, mitochondria

## Abstract

Sepsis is defined as “life-threatening organ dysfunction caused by a dysregulated host response to infection”. Contrary to the older definitions, the current one not only focuses on inflammation, but points to systemic disturbances in homeostasis, including metabolism. Sepsis leads to sepsis-induced dysfunction and mitochondrial damage, which is suggested as a major cause of cell metabolism disorders in these patients. The changes affect the metabolism of all macronutrients. The metabolism of all macronutrients is altered. A characteristic change in carbohydrate metabolism is the intensification of glycolysis, which in combination with the failure of entering pyruvate to the tricarboxylic acid cycle increases the formation of lactate. Sepsis also affects lipid metabolism—lipolysis in adipose tissue is upregulated, which leads to an increase in the level of fatty acids and triglycerides in the blood. At the same time, their use is disturbed, which may result in the accumulation of lipids and their toxic metabolites. Changes in the metabolism of ketone bodies and amino acids have also been described. Metabolic disorders in sepsis are an important area of research, both for their potential role as a target for future therapies (metabolic resuscitation) and for optimizing the current treatment, such as clinical nutrition.

## 1. Introduction

According to the latest standards, sepsis is defined as “life-threatening organ dysfunction caused by a dysregulated host response to infection” [1]. Contrary to the older definitions, the current one not only focuses on inflammation, but points to systemic disturbances in homeostasis, including metabolism. The term “metabolism” is defined as the totality of an organism’s chemical reactions. It consists of anabolic and catabolic processes that are physiologically in balance. This balance can be disturbed in pathological conditions such as sepsis. While the occurrence of a metabolic disorder in sepsis has been known for a long time, for many years, the efforts of scientists trying to develop a “cure for sepsis” have focused on inflammation. Despite their work, the current therapy of patients with sepsis consists mainly in antimicrobial treatment and supporting the functions of failing organs. Due to the fact that an innovative approach to the treatment of sepsis constitutes a great unmet need of modern medicine, it seems justified to pay attention to other components of the “dysregulated host response to infection”, which include metabolism, but also hemostasis, microbiota, thermoregulation and circadian rhythm [2,3]. Since understanding the role and place of metabolic disorders in the pathophysioslogy of sepsis can allow better management of treatment in sepsis patients, as well as find targets for potential new therapies, the aim of this review is to analyze current knowledge on sepsis metabolic disorders.

## 2. Outline of the Pathogenesis of Sepsis

**Figure 1:**
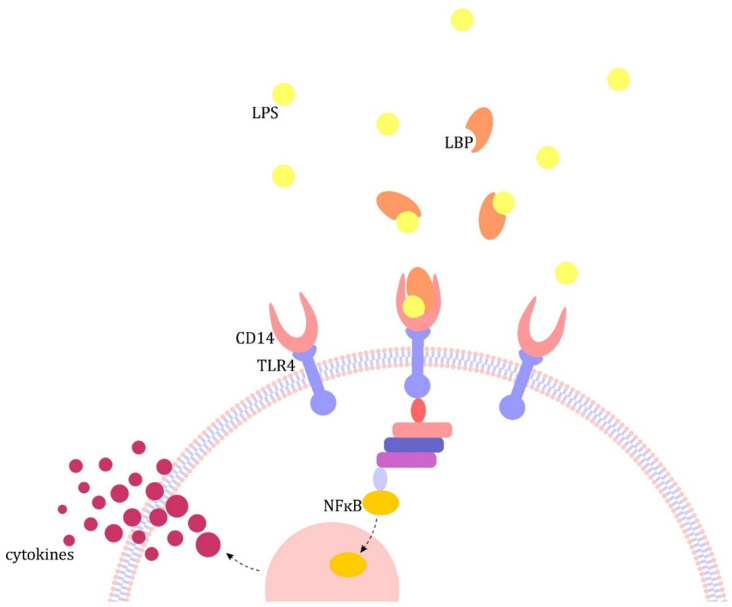
Pathogenesis of sepsis on the example of lipopolysaccharide (LPS) [4]. The major pathomechanism is the formation of complexes in the blood with lipopolysaccharide binding protein (LBP), which in turn binds to CD14 receptors present on monocytes/macrophages and neutrophils and circulating in the plasma. The resulting complex activates Toll-like receptors (TLRs), which transmit the signal inside the cell, leading to the translocation of the nuclear factor kB (NF-kB) to the cell nucleus and activation of pro-inflammatory cytokine gene promoters, including interleukin (IL) 1 and tumor necrosis factor α (TNF-α) [9].

An outline of the pathogenesis of sepsis and a detailed description of endocrine disorders occurring in its course have been described in another article by the authors. A schematic outline of the pathogenesis is shown in Figure 1, taken from the above-mentioned publication [4]. At this point, we would only like to emphasize the role of the cytokine storm in the pathogenesis of sepsis. The most important pro-inflammatory cytokines are interleukin 1 (IL-1), IL-6 and tumor necrosis factor α (TNF-α). They contribute to, inter alia, the development of a procoagulant state, increased production of reactive oxygen species (ROS) and nitric oxide (NO). The compensatory activation of anti-inflammatory mediators (e.g., IL-4, IL-10, IL-13) is also important. After varying time, the pathways for the synthesis of pro-inflammatory mediators may be exhausted and the immune balance may shift towards immunosuppression, characterized by impaired function of immune cells [5,6,7,8].

## 3. Metabolic Disorders in Sepsis

As mentioned in the introduction, changes in metabolism can be considered a typical disorder in the course of sepsis. The typical symptoms in sepsis patients such as fever, increased heart rate and respiratory rate, and activation of the immune system increase energy expenditure. On the other hand, metabolic disorders at the cellular level are described. Moreover, in sepsis, changes occur in the endocrine system and in the autonomic nervous system, discussed elsewhere [4]. The activation of these systems, called the neuroendocrine reaction, is dynamic and has a significant effect on metabolism [10]. It is believed that the promoters of these changes are mainly the pro-inflammatory cytokines—IL-1, IL-6 and TNF-α [11,12]. These cytokines play an essential role in the activation of the neuroendocrine response, which mediates metabolic changes and may also directly alter metabolism [10,13].

In the course of sepsis, an initial acute, hypermetabolic phase and the subsequent hypometabolic phase can be observed. The initial acute phase is characterized by a strong inflammatory response and the associated increase in catabolic processes, with the simultaneous inactivation of anabolic pathways. Its adaptive role is suggested—increased release of anterior pituitary hormones and insulin resistance contribute to the availability of substrates for energy production [14,15,16]. The later hypometabolic phase can also be adaptive—according to some authors, cells reach a state comparable to hibernation, which may protect them against adenosine triphosphate (ATP) depletion [8,17,18,19]. These changes lead to dysregulation of the metabolism of all macronutrients—carbohydrates, lipids and proteins [10]. Irahara et al. demonstrated a shift from glucose metabolism to lipid metabolism in a mouse sepsis model with low protein metabolism—these changes depended on the severity of sepsis. It was noted that plasma lipid levels decreased and liver lipid levels increased, suggesting that lipids were transported to the liver as an energy source [20]. Other studies describe a shift in metabolism towards “the stress state”, which occurs under the influence of cytokines and catecholamines. It includes the activation of hepatic gluconeogenesis and glycogenolysis, and systemic insulin resistance, leading to hyperglycaemia. An increase in hepatic lipolysis, leading to an increase in the concentration of plasma free fatty acids (FFA), and proteolysis, causing the release of amino acids from, inter alia, skeletal muscles, were also observed [21,22]. The result of these changes is hypercatabolism, causing the consumption not only of the body’s physiological reserves, but also the breakdown of proteins that play a structural or motor role [10]. There is a term “septic autocannibalism” in the literature, which reflects the nature of metabolism in patients with this condition [21,23]. Spanaki et al. demonstrated the presence of numerous correlations between amino acids, bioenergetics, metabolic indices, metabolites involved in the oxidative challenge, heat shock protein and metabolic hormones, suggesting the role of these molecules in the processes leading to the clinical manifestation of sepsis. The authors argue that “sepsis might mainly represent an energy pathway failure in the face of an acute infection-related organ failure” [24].

### 3.1. Mitochondrial Dysfunction

**Figure 2:**
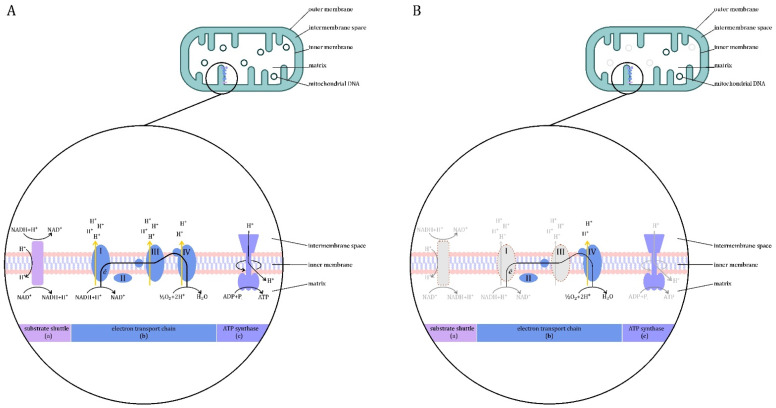
Production of energy in the mitochondria. (**A**) Physiological conditions. Mitochondria are formed from the permeable outer membrane, the intermembrane space, the selectively permeable inner membrane and the matrix. In the inner membrane, there are electron transport chain enzymes and ATP synthase. The tricarboxylic acid (TCA) cycle and β-oxidation enzymes are contained in the mitochondrion. The energy released during the oxidation of energy substrates is available inside the mitochondria in the form of reducing equivalents. The electron transport chain (ETC) (b) collects and transports the reducing equivalents, directing them to react with oxygen to form water. ETC is made up of four large protein complexes embedded in the inner mitochondrial membrane. The flow of electrons through the respiratory chain is driven by the redox potential difference and is mediated by three of the complexes (I, III, IV), substrates having a more positive potential than NAD+/NADH transfer electrons to complex III via complex II (instead of I). The flow of electrons through complexes I, III and IV causes the displacement of protons through the inner mitochondrial membrane—from the matrix to the intermembrane space. This allows the creation of a proton gradient that drives the synthesis of ATP. Proton-motive force (PMF), created by ions accumulating in the mesothelial space, is used by the ATP synthase (c) located in the inner mitochondrial membrane—it attaches a phosphate residue to adenosine diphosphate, creating ATP [31]. The inner mitochondrial membrane is impermeable to nicotinamide adenine dinucleotide (NADH), which is continuously produced in the cytosol by the glycolytic pathway. The transfer of reducing equivalents from NADH across the inner mitochondrial membrane requires the presence of a pair of dehydrogenase-conjugated substrates on both sides of the membrane, a system known as a substrate shuttles. Two types of substrate shuttles are involved in the transfer of reducing equivalents across the inner mitochondrial membrane—the glycerophosphate shuttle and the malate-aspartate shuttle (a) [31]. (**B**) Sepsis. In the course of sepsis, the expression and activity of some electron transport chain (ETC) complexes (b) are reduced. This may reduce the efficiency of ETC, whose role is to generate a proton gradient that drives the synthesis of ATP (c). Loss of some copies of mitochondrial DNA (mtDNA), which contains information on key subunits of the ECT complexes, has also been reported. In addition, sepsis can impair the operation of the substrate shuttle. Legend: gray color—insufficient ETC complexes and disturbed cellular processes.

Physiologically, most of the energy in the cells of the human body is produced in the mitochondria by oxidative phosphorylation [25]. They produce approximately 95% of the ATP consumed by cells and use approximately 90% of the oxygen taken up by the body [15]. Although ATP production is a key role for mitochondria, they are also involved in many other cellular processes, including cell death pathway, redox signaling, calcium flux and steroidogenesis [26,27,28,29,30]. The mitochondrial process of producing ATP is called oxidative phosphorylation. Its course under physiological conditions is shown schematically in Figure 2A.

Sepsis-induced dysfunction and mitochondrial damage is a major cause of cell metabolism disorders in sepsis (Figure 2B) [32]. Several possible mechanisms of this phenomenon have been described, including: mitochondrial damage by ROS, alteration of mitochondrial protein expression in a mechanism induced by pro-inflammatory cytokines, inhibition of key enzymes in the tricarboxylic acid (TCA) cycle, inhibition of mitochondrial enzyme complexes, and instability of the pyruvate dehydrogenase complex (PDC) [19,33]. 

In the course of sepsis, the concentration of ROS, nitric oxide (NO) and carbon monoxide in the cells increases. These substances interfere with some complexes of the mitochondrial electron transport chain (ETC), thus affecting cellular respiration [18,34,35]. The transcription of genes coding for proteins necessary for mitochondrial respiration is reduced. In sepsis patients, decreased expression of ETC complexes is observed [19,36,37,38]. A study using an experimental sepsis model has reported the loss of some copies of mitochondrial DNA (mtDNA) under the influence of oxidative stress and a decrease in the metabolic rate [39]. MtDNA contains information on the key subunits of ECT enzyme complexes, while information on structural subunits is encoded by nuclear DNA [40]. A reduction in the activity of complexes I and II has also been described, as well as a relationship between the phosphorylation of complex IV by inflammatory kinases and the exposure of cytochrome c and the initiation of apoptosis [41,42,43,44,45,46]. On the one hand, ETC dysfunction reduces its efficiency and, on the other hand, increases the risk of ROS formation. Some authors consider the transition to glycolysis as a protective–adaptive mechanism [47].

Organ failure in sepsis has previously been suggested to be related to insufficient oxygen and metabolic substrates to tissues. This theory was probably due to the frequent coexistence of shock (potentially impairing tissue perfusion) and lactic acidosis (supposedly due to hypoxia) in septic patients [48,49]. Although hypoxia is possible at the onset of sepsis, it has been shown that normal amounts of oxygen are delivered to the tissues during organ failure [50,51,52]. However, the tissue’s ability to use oxygen was limited, despite its availability in blood [53]. This gave rise to the theory that in sepsis cell metabolism is disturbed and oxygen utilization is impaired, despite its adequate availability. It has also been found that tissue oxygen consumption decreases in line with the increase in sepsis [19,54]. The phenomenon of the inability of cells to use oxygen has been called *cytopathic hypoxia* [34]. It is believed that initially cytopathic hypoxia may be adaptive and promote cell survival, but with duration and severity may contribute to organ failure [7,34]. 

Additionally, the hormonal changes that occur in the course of sepsis may contribute to a reduction in the ability of the oxidative phosphorylation pathway to produce ATP [17]. The key hormones affecting the efficiency of cellular metabolism include thyroid hormones, which have been shown to increase both the mitochondrial respiratory rate and the proton leak rate [55,56]. Whelan et al., in a study in a murine model of sepsis, showed a significant decrease in oxidative phosphorylation after 12 h of the experiment. There was also a significant increase in LPS-induced anaerobic respiration in hepatocytes. Metabolomic analysis showed a metabolic shift from carbohydrate-utilizing metabolism towards fatty acid and amino acid utilization [57].

Numerous studies have shown that there is a correlation between the energy efficiency of cells and the result of treatment. Patients who died showed lower levels of ATP in their muscles compared with those who survived [58,59]. A similar correlation has been found from studies of various tissues in animal models of sepsis [60,61].

### 3.2. Carbohydrate Metabolism

Deregulation of carbohydrate metabolism is the most characteristic feature of sepsis-related metabolic disorders. The most common clinical symptom is hyperglycemia. Its causes include altered glycogen metabolism and significant insulin resistance. The totality of the molecular events leading to this state is much more complex and extends to the action of proinflammatory cytokines, as discussed in the relevant sections of this article.

It has been suggested that hyperglycemia, in the course of sepsis, may be adaptive—an increase in blood glucose levels and limitation of its use by some tissues allows glucose to be redirected to the cells of the immune system, which enables them to meet their increased energy requirements [62]. It has been shown that in activated cells of the immune system, there is a shift in metabolism towards oxygen glycolysis (Warburg effect) [63]. According to other authors, potential recipients to whom increased blood glucose is dedicated may also include damaged tissues that may have ineffective mechanisms of mitochondrial respiration [64]. The initial, but transitory source of glucose in situations of increased demand is hepatic glycogen, which is broken down into glucose by glycogenolysis [64]. When glycogen reserves are lacking, the main source of glucose becomes gluconeogenesis—the process of converting non-carbohydrate compounds into glucose. The substrates for this process can be amino acids, lactate, glycerol, and propionate. Gluconeogenesis occurs in the liver and kidneys [65]. It is an energy-consuming process, but necessary, as it provides an energy substrate for glucose-dependent cells [66]. 

Glucose is transported to cells by glucose transporters, the so-called GLUT. GLUT1 is a transporter responsible for insulin-independent glucose uptake, it is found, inter alia, in the central nervous system (CNS) and erythrocytes. GLUT2 is bi-directional—it allows both glucose uptake and release—and is found in the liver, kidneys, small intestine, and pancreatic β-cells. GLUT4 is responsible for insulin-dependent glucose uptake and is found in heart, muscle and adipose tissue [64,65,67]. Studies in an animal sepsis model showed a 67% increase in glucose uptake compared to control and an almost twofold increase in GLUT1 expression in skeletal muscle, while GLUT4 expression remained unchanged [68]. GLUT1 upregulation increases insulin-independent glucose uptake [69,70].

Maintaining a constant blood glucose concentration is one of the most precisely regulated homeostatic mechanisms. The liver, extrahepatic tissues and hormones play a role in this process. Physiologically, blood glucose readily penetrates the liver cells and pancreatic β cells (GLUT2), while its penetration into most other cells is hormonally regulated. The β cells of the pancreatic islets produce insulin, which physiologically plays a major role in glycemic control. Insulin has a hypoglycemic effect, increasing the transport of glucose from the blood to adipose tissue and muscle cells—the basic mechanism is the translocation of the GLUT4 transporter from the inside of these cells to their cell membrane. In addition, insulin acts to regulate the enzymes that control glycolysis, gluconeogenesis and glycogenesis, and it influences the metabolism of fatty acids and proteins. The other hormones that influence blood glucose levels are hyperglycemic, they are called anti-regulatory hormones. These include glucagon, cortisol, growth hormone (GH), and catecholamines. Numerous cytokines are also antagonistic to insulin [65].

Insulin resistance is defined as the failure of anabolic processes to respond to the normal action of insulin and is one of the major metabolic changes in sepsis [71,72]. In the course of sepsis, potential mechanisms leading to this phenomenon include an increase in the concentration of counter-regulatory hormones in the plasma, activation of the sympathetic nervous system, and direct effect of cytokines on tissues [17,73]. In sepsis, insulin resistance increases hepatic glucose production as a result of glycogenolysis and gluconeogenesis. Moreover, impaired insulin sensitivity of peripheral tissues shifts their metabolism towards lipolysis and proteolysis [64].

**Figure 3:**
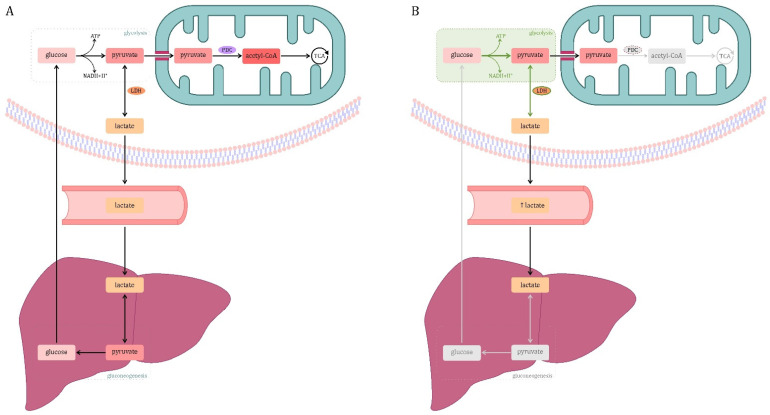
Glucose metabolism. (**A**) Physiological conditions. Glycolysis is the conversion of glucose into pyruvate. In some cases, for example, when glycolysis occurs anaerobically or in cells incapable of pyruvate oxidation, pyruvate may be converted to lactate by the enzyme lactate dehydrogenase (LDH). Blood lactate (mainly formed in skeletal muscle) is taken up by the liver, converted to pyruvate and incorporated into the gluconeogenesis pathway (Cori cycle). In other cases, the pyruvate formed in the cytosol is transported to the mitochondria by a proton symporter. In the mitochondrion, pyruvate undergoes oxidative decarboxylation to acetyl-CoA mediated by the multi-enzyme pyruvate dehydrogenase complex (PDC). Oxidation of pyruvate to acetyl-CoA connects glycolysis with the tricarboxylic acid (TCA) cycle [74]. (**B**) Sepsis. This is due, inter alia, to a decrease in pyruvate dehydrogenase complex (PDC) activity. One explanation is the phosphorylation of the enzyme by pyruvate dehydrogenase kinases (PDKs) and its decreased expression in sepsis. Moreover, in sepsis, the hypoxia-inducible factor 1α (HIF-1α) is activated, which is one of the main mediators of glycolysis. HIF-1α regulates the expression of genes of enzymes related to glycolysis (hexokinase, phosphofructokinase-1, glucose-6-phosphate dehydrogenase, lactate dehydrogenase, pyruvate dehydrogenase kinase, glutamate transporter-1). The intensification of glycolysis in combination with the failure of entering pyruvate to the tricarboxylic acid (TCA) cycle increases the formation of lactate. A decrease in hepatic lactate-based gluconeogenesis has been reported. This increases the production of lactate, while blocking the main routes of its disposal, which results in an increase in its concentration in the blood. Legend: gray color—inefficient cellular processes; green color—increasing the activity of the process or enzyme.

Glycolysis is a metabolic pathway that takes place in the cytosol of all human cells, and its course under physiological conditions is shown in Figure 3A.

In sepsis, there is an increase in glycolysis activity (Figure 3B). It is an alternative (but less efficient than mitochondrial respiration) source of ATP. Despite the low ATP production efficiency, glycolysis provides substrates for the synthesis of amino acids, lipids, and nucleotides that are important in the metabolism of activated immune cells. The advantage of aerobic glycolysis is that energy is produced relatively quickly, which is very beneficial in an intense immune response [75,76]. The increase in glycolysis activity described in sepsis is due to a decrease in PDC activity and impaired mitochondrial function. It is also associated with a phenomenon known as cytopathic hypoxia. 

Significant reductions in PDC activity have been reported in the course of sepsis [77,78]. Phosphorylation of the enzyme by pyruvate dehydrogenase kinases (PDKs) (PDK1-PDK4) has been proposed as a possible cause. PDKs are enzymes regulated by transcription factors, such as: hypoxia-inducible factor 1α (HIF-1α), glucocorticoid receptor (GR) and peroxisome proliferator-activated receptor (PPAR-α) [79]. Studies describing the reduction of PDC expression in sepsis are also available [19,36,80]. 

HIF-1α is a transcription factor sensitive to oxygen concentration in the cellular environment, and it is one of the main mediators of glycolysis. HIF-1α regulates the expression of genes of enzymes related to glycolysis (hexokinase, phosphofructokinase-1, glucose-6-phosphate dehydrogenase, lactate dehydrogenase, pyruvate dehydrogenase kinase, glutamate transporter-1), as well as those related to inflammation, e.g., IL-1β and inducible synthase nitric oxide (iNOS) [76,81]. Under normoxic conditions, HIF-1α is produced continuously and, due to its short half-life (5 min), it is degraded by the 26S proteasome. Tissue hypoxia induces an increase in the production of ROS in the mitochondria (low oxygen concentration reduces the efficiency of ATP production, resulting in increased proton leakage and ROS production) [82]. ROS induce inhibition of HIF1α targeting to the proteasome, which increases its stability [47]. HIF determines the adaptive response to hypoxia, increases the supply and reduces oxygen consumption. Its adaptive effect in acute phase involves a shift from mitochondrial respiration to glycolysis [83]. Interestingly, bacteria can increase the expression of the HIF1α protein even under normoxic conditions. It has been shown that LPS can promote the transcription of the HIF1α gene in monocytes and macrophages in a NFκB dependent mechanism [84,85,86]. The ability to induce HIF1α expression under normoxic conditions has also been demonstrated for TNFα [87]. Another important mediator of the process of glycolysis is pyruvate kinase isoenzyme M2 (PKM2). This enzyme is a coactivator of HIF-1α and participates in the final stage of glycolysis (dephosphorylation of phosphoenolpyruvate to pyruvate). PKM2 is induced by hypoxia and LPS [41,88].

A century ago, biochemist Otto Warburg noticed that cancer cells produce energy through glycolysis, rather than using much more efficient oxidative phosphorylation [89]. He believed that the cause was the irreversible inactivation of mitochondria, which is now known to be a rare phenomenon [90]. Current knowledge allows us to conclude that the Warburg effect is a complex phenomenon regulated by many metabolic factors. Moreover, its occurrence has been shown not only in neoplastic cells, but also in sepsis [91].

In sepsis, cells respond with a hypoxic response despite the absence of hypoxia. The transcription and activation of HIF1-α, as described above, are increased. This transcription factor induces genes encoding glycolysis proteins—hexokinase, phosprofructokinase-1 and lactate dehydrogenase [76,81]. Lactate is transported in the blood and may serve as an energy substrate in some tissues. Under physiological conditions, blood lactate (mainly formed in skeletal muscle) is taken up by the liver, converted to pyruvate and used in the gluconeogenesis pathway (Cori cycle) (Figure 3A). However, in an experimental sepsis model, significant reductions in hepatic lactate-based gluconeogenesis have been reported (Figure 3B) [92]. This may be related to the impairment of the shuttle systems across the inner mitochondrial membrane in sepsis, which play an important role in this process by transporting protons from lactate oxidation to the mitochondria [93,94]. This causes an increase in production, while blocking the main routes of lactate disposal, which results in an increase in its concentration in the blood. Therefore, lactate may be used as one of the prognostic markers of sepsis—its concentration in the blood correlates with the severity of sepsis and mortality [95,96]. Lactate, as an anion of lactic acid, when accumulated, can lead to lactic acidosis, which has both metabolic and clinical consequences. Its role in the modulation of inflammation has also been described—it has been shown to increase TLR4 (main LPS receptor) signaling and NF-κB mediated gene transcription [97].

Despite the characteristic hyperglycemia that occurs in patients with sepsis, they may also develop hypoglycemia later in the disease. However, while hypoglycemia is a constant phenomenon in animal models of sepsis, it is not so common in humans [98,99,100].

The processes leading to hypoglycemia in sepsis are poorly understood. Possible causes include depletion of glycogen reserves, increased peripheral glucose consumption, decreased gluconeogenesis and decreased nutrient supply [101,102,103]. There are studies showing a beneficial role for the metabolic state induced by fasting in bacterial sepsis. The metabolic changes caused by prolonged fasting include hypoglycemia, lipolysis and ketogenesis. Ketone bodies are involved in pathways that protect against high concentrations of ROS [103]. However, hepatic gluconeogenesis has been shown to be a necessary pathway to avoid fatal hypoglycemia in response to acute bacterial infection [102]. This indicates that despite the potentially beneficial role of hypoglycemia, blood glucose levels must be kept within the physiological range [104].

Both very high and very low blood glucose levels have been shown to correlate with poorer outcome in patients with sepsis [105,106,107]. Severe hyperglycemia can cause liver and kidney damage, endothelial dysfunction, mitochondrial damage and neurocognitive disorders, while severe hypoglycemia can cause cardiac arrest, neurocognitive and hemostatic disorders [105,108]. In the early 2000s, randomized controlled trials showed the promising effects of insulin glycemic control in patients with sepsis [109,110,111]. Based on these studies, tight glycemic control (TGC) was introduced into the standard of care for sepsis. However, subsequent studies not only failed to confirm its efficacy in reducing mortality, but also showed that TGC significantly increases the risk of hypoglycemia in patients or even increases mortality [112,113]. However, it should be noted that in later studies, the control group was usually treated with insulin, with a broader blood glucose tolerance, while in the first RCTs, the control group’s glycemia was not controlled. 

### 3.3. Lipid Metabolism

**Figure 4:**
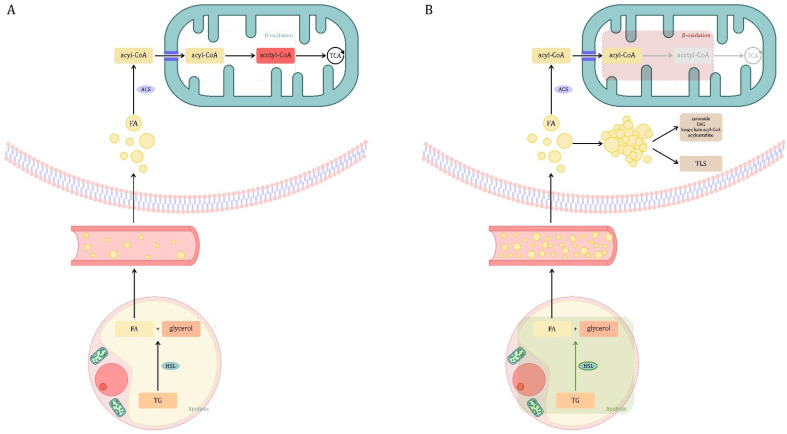
Lipid metabolism. (**A**) Physiological conditions. Triglycerides (TG) stored in adipocytes must undergo a three-step process by which other tissues can access their stored energy reserves. The first step, lipid mobilization, involves lipolysis of TG to fatty acids (FA) and glycerol, which diffuse into the plasma and are transported to the target tissues. TG is lipolyzed by the enzyme hormone-sensitive lipase (HSL). This process is influenced by many hormones. Insulin inhibits the action of HSL. Lipolysis-promoting hormones include catecholamines, glucagon, adrenocorticotropic hormone (ACTH), thyroid-stimulating hormone (TSH), growth hormone (GH) and vasopressin. Moreover, most lipolytic processes require the presence of thyroid hormones and glucocorticosteroids for optimal effect [114]. The second step is to activate FA and transport them to the mitochondria. The enzyme acyl-CoA synthase catalyzes the conversion of FA to active fatty acid (acyl-CoA). Then, long-chain acyl-CoA molecules penetrate the inner mitochondrial membrane as carnitine derivatives (acylcarnitine). The third step is β-oxidation—acyl-CoA molecules are broken down into acetyl-CoA, which can be oxidized in the tricarboxylic acid (TCA) cycle [115]. (**B**) Sepsis. In the initial stage of sepsis, lipolysis in adipose tissue is upregulated. The role of hormone-sensitive lipase (HSL) activation under the influence of LPS and prolipolytic hormones is suggested. Insulin resistance also contributes to the activation of lipolysis. Patients with sepsis have increased levels of fatty acids (FA) and triglycerides (TG) in the blood. This may be due to insulin resistance in the liver and adipose tissue. In the course of sepsis, the β-oxidation process is disturbed, which may be associated with a decrease in the expression of genes belonging to the PPAR-α signaling pathway. Additionally, the transport of long-chain FA to the mitochondria may be impaired due to possible accumulation of malonyl-CoA generated from glucose in hepatocytes. The lower efficiency of β-oxidation can cause energy deficiency, lipotoxicity and mitochondrial dysfunction, leading to organ damage. Lipotoxicity is a pathological metabolic phenomenon resulting from the accumulation of lipid intermediates in tissue other than adipose tissue. Lipid accumulation may lead to some of their metabolites (e.g., diacylglycerol (DAG), ceramide) reaching levels potentially harmful to cells. It is believed that excess lipids can be diverted to non-oxidative pathways and be transformed into toxic lipid species (TLS), which can damage mitochondria, modify cell signaling and increase apoptosis (lipoapoptosis). Legend: gray color—inefficient cellular processes; green color—increasing the activity of the process or enzyme; red color—lowering the activity of the process or enzyme.

Triglycerides (TG) accumulated in the cytoplasm of fat cells constitute the largest store of energy substrates in the human body. For this energy to be used by other tissues, TG must pass a three-stage process. It is presented in Figure 4A.

During the initial stage of sepsis, lipolysis in adipose tissue is upregulated (Figure 4B) [116,117,118,119]. The mechanism of this phenomenon has not been fully understood. One study showed that LPS caused an increase in the phosphorylation of hormone-sensitive lipase (HSL) in Ser650—a modification known as enzyme activating [117]. It was also found that the administration of LPS increases the level of cAMP-dependent protein kinase A (PKA)-dependent phosphorylation of perilipin 1 (PLIN1), which also promotes the activation of lipolysis [117,120]. In addition, it is known that sepsis increases the concentration of prolipolytic hormones—catecholamines, growth hormone (GH), glucagon and cortisol. Additionally, insulin resistance, common in sepsis patients, promotes lipolysis—insulin is an inhibitor of this process [120]. In sepsis, there is an increase in the concentration of fatty acids (FA) and TG in the blood—it has been found that their plasma levels in sepsis patients are up to four times higher than in healthy subjects [121,122,123,124,125]. In contrast, the data on the levels of circulating lipoproteins are varied. Reduction in low (LDL) and high-density (HDL) lipoproteins has been reported, which is interesting as there is evidence of their ability to neutralize LPS and have anti-inflammatory effects. In contrast, an increase in very low-density lipoprotein (VLDL) has been demonstrated [64,121]. The changes described may be caused by the insulin resistance of the liver and adipose tissue [17,122]. Additionally, LPS and proinflammatory cytokines can induce hepatic TG synthesis and de novo synthesis of FA [126,127].

The oxidation of FA is a key source of energy in conditions of increased demand, including sepsis [128,129]. However, studies suggest that in the course of sepsis, despite increased lipolysis, the use of FA as an energy substrate is disturbed [64]. Peroxisome proliferator-activated α receptor (PPAR-α) is the main transcription factor regulating the expression of genes encoding proteins involved in the β-oxidation pathway [130]. In patients with septic shock, significant reduction in the expression of genes belonging to the PPAR-α signaling pathway has been described [131]. LPS has also been shown to reduce the expression of PPAR-α and the co-activator PPAR-γ 1α (PGC-1α), which is the major transcription co-factor of PPAR-α [132]. These changes lead to decreased expression of PPAR-α target genes, which may disrupt the β-oxidation process. The lower efficiency of β-oxidation can cause organ damage, causing energy deficiency, lipotoxicity and mitochondrial dysfunction [41].

The phenomenon of mutual competitive regulation of glucose and FA oxidation, called the Randle cycle (or the glucose-fatty acid cycle) is also known. Randle et al. showed that the use of one nutrient directly (without hormonal mediation) inhibits the use of the other. Acetyl-CoA, a product of β-oxidation of FA, inhibits the activity of PDC, and citrate (the product of the first reaction of the TCA cycle after acetyl-CoA entry) inhibits phosphofructokinase, a key enzyme in the glycolysis process [133]. On the other hand, glucose in hepatocytes can be converted to malonyl-CoA, which inhibits long-chain FA transport into the mitochondria. Accumulation of malonyl-CoA may inhibit hepatic metabolism of FA, increasing their concentration in the liver and blood [64].

As described, in the course of sepsis, lipolysis is intensified, and at the same time disturbances of β-oxidation may occur. This situation may lead to FFA accumulation and lipotoxicity [41]. Lipotoxicity is a pathological metabolic phenomenon resulting from the accumulation of lipid intermediates in tissue other than adipose tissue that can cause cell damage [134]. Clinically, the accumulation of fat in the parenchymal organs is called steatosis and has been identified in the liver, kidney and heart muscle in sepsis [135,136,137]. Lipid accumulation may lead to some of their metabolites (e.g., diacylglycerol, ceramide, saturated fatty acids) reaching critical levels, potentially harmful to cells [138]. It is believed that excess lipids can be diverted to non-oxidative pathways and transformed into toxic lipid species (TLS), which can damage mitochondria, modify cell signaling and increase apoptosis (lipoapoptosis) [41,138,139,140,141]. TLS may also be involved in the induction of ferroptosis, iron-dependent programmed cell death, but the role of ferroptosis in sepsis has not yet been investigated [142,143,144].

### 3.4. Ketone Metabolism

The metabolic changes in sepsis can lead to a response similar to prolonged fasting—increased lipolysis and ketogenesis [103]. Ketone bodies—acetoacetate, 3-hydroxybutyrate and acetone—are formed in the liver mitochondria during intense FA oxidation, and their synthesis is tightly controlled by PPAR-α. Ketone bodies can be an energy substrate in extrahepatic tissues, and they are an important source of energy for the brain and skeletal muscles in conditions of limited glucose availability [115,145]. Ketogenesis allows tissues with a low ability to oxidize free FA to use energy from the reserves accumulated in adipose tissue. In addition, ketone bodies are involved in pathways that protect against high concentrations of ROS. A study in mice deficient in PPAR-α showed that LPS-induced endotoxemia in the absence of ketogenesis was fatal [103]. Ketogenesis appears to be a valuable metabolic pathway in sepsis; however, due to the PPAR-α related disorders described above, it may be inadequately active.

### 3.5. Amino Acid Metabolism

Although proteins are not a classic energy substrate, amino acids (AA) can be catabolized to amphibole intermediates that can serve as an energy source or participate in the biosynthesis of lipids and carbohydrates. The release of amino acids from proteins occurs in the process of proteolysis—hydrolysis of the peptide bond catalyzed by proteases. Sepsis is characterized by catabolism, which leads not only to the use of physiological reserves of energy substrates such as fats and carbohydrates, but also to the breakdown of proteins. It was found that patients with sepsis lost 13% of their body protein within 3 weeks of disease (despite intensive therapy including nutritional therapy) [146]. Proteolysis is best documented in skeletal muscle [41]. In the studies of the metabolic response of skeletal muscles to sepsis, inhibition of protein synthesis and activation of their proteolysis have been demonstrated [147,148,149,150]. The most abundant released AA are alanine and glutamine, which, according to some authors, proves their de novo synthesis [151,152]. Altered concentrations of circulating AA are also observed in sepsis [153,154].

The signaling that induces proteolysis in sepsis is poorly described. A major role is assigned to hormone signaling, especially glucocorticosteroids (GCs) and glucagon [41,64]. Blocking GCs has been shown to attenuate proteolysis [155]. Other factors, such as insulin resistance and acidosis, may also play a role [156,157]. Among the causes of proteolysis in the course of sepsis are reshuffling of energy-rich molecules and increased liver demand for AA, related to the acute phase response [41]. Under physiological conditions, the liver mainly synthesizes constitutive proteins, such as albumin and transferrin, while in sepsis, synthesis changes from constitutive proteins to acute-phase proteins, which include procalcitonin, C-reactive protein, complement factors, haptoglobin, α2-macroglobulin and α1-acid glycoprotein [12,158]. 

AA released into the bloodstream is used by the liver for gluconeogenesis and acute phase protein synthesis [159,160]. There is also evidence of the use of glutamine as an energy substrate by enterocytes and polymorphonuclear neutrophils [161,162,163]. Branched chain amino acids (BCAA)—valine, leucine and isoleucine—can act as an acceptor of the ketone groups of pyruvate and glutamate, which leads to the formation of glutamine and alanine. Glutamine and alanine can be used as energy substrates by the liver, intestines and kidneys. In addition, some AA, such as glutamine, can be involved in the production of cytokines and acids, supporting activated cells of the immune system. Arginine can also be converted into NO released by macrophages [41].

The increased protein breakdown observed in sepsis leads to a negative nitrogen balance, a state where nitrogen excretion exceeds nitrogen consumption, which is typical of wasting [21]. Catabolism without proper nutritional support solving the problem of negative nitrogen balance can lead to the loss of up to 1 kg of muscle tissue per day [66]. Loss of lean body mass in sepsis patients has also been shown to be a risk factor for worse treatment outcome and increased mortality [164]. A separate risk factor may be the coexistence of increased proteolysis and hepatic failure, leading to an increase in blood ammonia concentration (due to urea cycle failure), which may cause serious complications such as hepatic encephalopathy [165,166].

## 4. Conclusions

Energy metabolism in sepsis is clearly disturbed. The changes seem to affect the metabolic pathways of all energy substrates and contribute to the pathogenesis of this state. As stated by Singer et al., a more sophisticated understanding of the sequence, dynamics and interaction between the metabolic, hormonal and immunological changes occurring would provide a logical basis for patient-tailored therapeutic interventions [17]. The adaptive role of some of the described changes as postulated by many authors is noteworthy. Counteracting changes of adaptive importance could have a detrimental effect. It has been suggested that whether a disorder is adaptive or harmful is decided not by its nature, but by its duration and severity [7]. Therefore, the timing and intensity of potential therapeutic intervention seem to be of key importance [17]. Additionally, adequate clinical nutrition requires us to be aware of the dynamics and diversity of metabolic disorders in the course of sepsis. Studies have shown that both insufficient and excessive energy supply are associated with worse treatment outcomes [167]. This indicates the need for careful nutrition planning and the validity of using energy expenditure measurement, with indirect calorimetry as the gold standard [168], in this group of patients.

Due to both energetic and metabolic consequences, mitochondrial dysfunction appears to play a significant role in the pathogenesis of sepsis. This makes them a valuable subject for further research and a potential target for future therapies. There are studies showing that metabolic resuscitation could prove to be a breakthrough in the treatment of sepsis [169]. Other drugs that can potentially alleviate metabolic disorders, such as β-blockers and poly (ADP-ribose) polymerase (PARP) inhibitors, are also being investigated [170,171]. In a study published in 2018, Van Wyngene et al. proposed three metabolic pathways, the potential modulation of which could be both safe and effective in sepsis patients—dependent on increased HIF-stimulated glycolysis lactate production, dependent on impaired β-oxidation accumulation of FFA in the blood and the inadequate efficiency of hepatic ketogenesis [41]. Since the possibility of appropriate treatment for these disorders could address an unmet need for a “cure for sepsis”, this issue undoubtedly deserves further researches.
